# Validation and Reliability of a Smartphone Application for the International Prostate Symptom Score Questionnaire: A Randomized Repeated Measures Crossover Study

**DOI:** 10.2196/jmir.3042

**Published:** 2014-02-10

**Authors:** Jae Heon Kim, Soon-Sun Kwon, Sung Ryul Shim, Hwa Yeon Sun, Young Myoung Ko, Dong-Il Chun, Won Jae Yang, Yun Seob Song

**Affiliations:** ^1^Department of Urology, Soonchunhyang UniversityCollege of MedicineSoonchunhyang University HospitalSeoulKorea, Republic Of; ^2^Biomedical Research InstituteSeoul National University Bundang HospitalSeongnamKorea, Republic Of; ^3^Department of Epidemiology and Medical InformaticsKorea UniversitySeoulKorea, Republic Of; ^4^Department of Industrial and Management EngineeringPohang University of Science and TechnologyPohangKorea, Republic Of; ^5^Department of Orthopaedics, Soonchunhyang UniversityCollege of MedicineSoonchunhyang University HospitalSeoulKorea, Republic Of

**Keywords:** smartphone, International Prostate Symptom Score, lower urinary tract symptoms, health survey, questionnaires

## Abstract

**Background:**

Smartphone-based assessment may be a useful diagnostic and monitoring tool for patients. There have been many attempts to create a smartphone diagnostic tool for clinical use in various medical fields but few have demonstrated scientific validity.

**Objective:**

The purpose of this study was to develop a smartphone application of the International Prostate Symptom Score (IPSS) and to demonstrate its validity and reliability.

**Methods:**

From June 2012 to May 2013, a total of 1581 male participants (≥40 years old), with or without lower urinary tract symptoms (LUTS), visited our urology clinic via the health improvement center at Soonchunhyang University Hospital (Republic of Korea) and were enrolled in this study. A randomized repeated measures crossover design was employed using a smartphone application of the IPSS and the conventional paper form of the IPSS. Paired *t* test under a hypothesis of non-inferior trial was conducted. For the reliability test, the intraclass correlation coefficient (ICC) was measured.

**Results:**

The total score of the IPSS (*P*=.289) and each item of the IPSS (*P*=.157-1.000) showed no differences between the paper version and the smartphone version of the IPSS. The mild, moderate, and severe LUTS groups showed no differences between the two versions of the IPSS. A significant correlation was noted in the total group (ICC=.935, *P*<.001). The mild, moderate, and severe LUTS groups also showed significant correlations (ICC=.616, .549, and .548 respectively, all *P*<.001).There was selection bias in this study, as only participants who had smartphones could participate.

**Conclusions:**

The validity and reliability of the smartphone application version were comparable to the conventional paper version of the IPSS. The smartphone application of the IPSS could be an effective method for measuring lower urinary tract symptoms.

## Introduction

Lower urinary tract symptoms (LUTS) suggestive of benign prostatic hyperplasia (BPH) affect a majority of men and show an increasing prevalence with aging [1]. Male patients with LUTS suffer from significant impairment of their quality of life (QOL) and interference in daily living activities due to urinary dysfunction [2].

The primary treatment goal for men with clinical manifestations of BPH is to reduce or relieve LUTS. Therefore, the measurement of LUTS is a key factor in the evaluation of these patients, both in clinical practice and in research studies [2,3].

The International Prostate Symptom Score (IPSS) is the most widely used diagnostic tool in urology and is widely available, validated, and has been translated into many languages [3]. The IPSS scale correlates positively and significantly with global measures of difficulty and health complications associated with LUTS [4-6].

To date, the paper-based IPSS has been used worldwide. Although paper-based questionnaires have been the standard tools for screening or monitoring of medical conditions, this method has several problems, including data collection and entry errors [7].

Over the past 20 years, questionnaires have been developed using electronic systems, such as Web surveys on personal computers or personal digital assistants, and more recently using smartphones. There has been a meteoric rise in the use of smartphones, which has reached approximately 6 billion people worldwide [8], enabling smartphones to function as new tools for measuring the health of individuals.

Most smartphone applications have focused on education and communication for medical school students and clinicians [9]. However, several smartphone application questionnaires have been introduced in clinical use, including psychiatry and sleep disorders [10-12].Considering the worldwide use of the IPSS in clinical fields, a smartphone application of the IPSS could be very popular, both for patients and clinicians.

The ultimate goal of smartphone-based assessment is the establishment of a home diagnostic device that enables easy screening and monitoring of a disease by scoring data and thus reducing the time to diagnosis and treatment, as well as overall costs. Most questionnaires are originally designed as paper-based questionnaires and, therefore, validation of a smartphone-based version is required due to the possibility of response bias between paper and smartphone versions [13].The aim of this study was to examine the validity and reliability of a smartphone application version of the IPSS by quantitative analysis and to show the satisfaction rate compared with the conventional paper version of the IPSS.

## Methods

### Study Sample

From June 2012 to May 2013, 1581 male patients presenting with LUTS (≥ 40 years old) visited our urology clinic via the health improvement center at Soonchunhyang University Hospital (Republic of Korea) and were enrolled in this study. All patients underwent a complete history, physical examination, and urinalysis, and completed an IPSS questionnaire. Patients who had a history of cancer of any organ, neurologic diseases or disorders, uncontrolled hypertension or diabetes, psychiatric disorders, prostatic surgery, liver cirrhosis, or renal failure were excluded from this study. Participant data were recorded in a prospective database and the study was approved by the Institutional Review Board of Soonchunhyang University Hospital.

### Methodology

A randomized repeated measures crossover design was employed using the smartphone application and conventional paper form of the IPSS. One investigator conducted face-to-face interviews with all study participants, using structured explanations. The Korean version of the IPSS has been verified for relevance and reliability, and is the most popular diagnostic instrument for LUTS in Korea [14]. There was a 1-week break before and after completion of the alternative versions of the IPSS in order to reduce carryover effects. Questionnaires were randomly assigned to the smartphone or paper version of the IPSS. The supervisors obtained oral informed consent from participants before the study.

### Developing the Smartphone Application of the IPSS

The smartphone application was specifically developed for Android model smartphones (Android is the operating system created by Google). While actively answering the IPSS in the smartphone application, participants could go backward to correct answers before they chose “save” to go on to the next question or to finish the test. The responses on the smartphone application of the IPSS were automatically transferred to the database where only supervisors could access the information.

### System Stability

For a successful study trial, the stability of the system providing the application of the IPSS should be safe and stable. To this end, the server manager closely monitored the system during the performance of this trial. Prior to this trial, we conducted a pilot test to check the stability of the smartphone application of the IPSS and the data collection server.

### Main Outcome for Validity

The overall hypothesis of this study was that participants would find both the smartphone application and paper version of the IPSS feasible and acceptable to use, which means that the smartphone application of the IPSS would not be inferior to the paper version of the IPSS. Validity was defined by non-difference in the total score of the IPSS and in each item of the IPSS.

### Main Outcome for Reliability

Reliability referred to the consistency of IPSS scores obtained by the same person between the paper and smartphone version. There were many statistics available to measure reliability. The reliability test was conducted by the intraclass correlation coefficient (ICC). ICC was used to measure reliability for the paper and smartphone IPSS scores and ranged from 0 (no agreement) to 1 (perfect agreement). Reliability was defined by correlation between the two versions of the IPSS.

### Delivery Method Compliance

In order to determine the compliance rate, the participation and preference rates were investigated by specific questions at the end of the trial: “Which method would you be willing to use to complete the assessment more easily?” and “Which method would you prefer?”

### Power Calculation

The base of our sample size was dependent on the hypothesis that a smartphone application of the IPSS was not inferior to the conventional paper-based IPSS [15]. We used an alpha error of .05 and beta error of .2. The calculated sample size was 980. Considering a 10% decline or withdrawal rate, the minimal sample size was calculated to be 1100.

### Data Analysis

We analyzed differences by paired *t* test for the two pairs of questionnaires. Reliability was assessed using ICC and a two-way random effect model, assuming a single measurement and absolute agreement. Statistical analyses were performed using SPSS version 20.0 for Windows. All statistics were two-tailed and *P* values <.05 were considered statistically significant.

### Preservation of Data Security

Individual or total scores were not accessible by participants through their smartphones. After completing the paper version of the IPSS, the data collector recorded the data after checking the data transfer error and then sent the data to the statistician. After completing the smartphone application of the IPSS, the data were transferred automatically to the special server and then sent to the statistician. After completing the analysis, all information regarding the IPSS was removed by the supervisor.

## Results

### Basic Characteristics of the Participants

**Figure 1:**
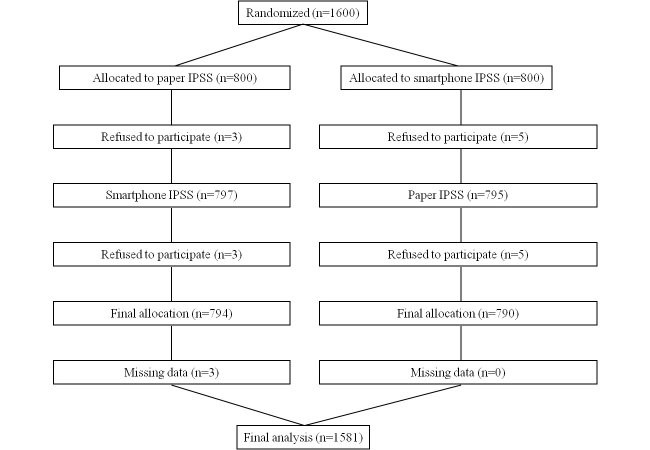
Flow chart of study participants.

The mean age of the participants was 58.49 (SD 7.22). The mean total score of the IPSS paper version was 11.04 (SD 7.76) and the mean total IPSS score of the smartphone version was 11.03 (SD 7.77). There were 668 (42.25%, 668/1581) subjects in the mild LUTS group, 643 (40.67%, 643/1581) subjects in the moderate LUTS group, and 270 (17.08%, 270/1581) subjects in the severe LUTS group (Table 1). The refusal rate in total was 16 and there were 3 cases of missing data, so the final allocation was 1581 cases (Figure 1).

The total rates of mild, moderate, and severe LUTS groups were significantly different (*P*<.001). In the comparison of the rates of these groups, the mild and moderate LUTS groups were not different (*P*=.471), but there were significant differences (*P*<.001) between the mild and severe LUTS groups and the moderate and severe LUTS groups.

**Table 1:** Baseline characteristics (n=1581).

	n	Mean (SD)	Range
Age	1581	58.49 (7.22)	40-79
Paper IPSS^a^ Q1	1580	1.69 (1.61)	0-5
Paper IPSS Q2	1581	1.57 (1.42)	0-5
Paper IPSS Q3	1579	1.63 (1.54)	0-5
Paper IPSS Q4	1579	1.32 (1.42)	0-5
Paper IPSS Q5	1581	2.30 (1.66)	0-5
Paper IPSS Q6	1580	1.27 (1.46)	0-5
Paper IPSS Q7	1581	1.28 (1.15)	0-7
Paper Total IPSS	1581	11.04 (7.77)	0-35
Paper QOL^b^	1546	2.77 (1.38)	0-6
Smartphone IPSS Q1	1580	1.68 (1.61)	0-5
Smartphone IPSS Q2	1581	1.57 (1.42)	0-5
Smartphone IPSS Q3	1579	1.63 (1.54)	0–5
Smartphone IPSS Q4	1579	1.32 (1.42)	0-5
Smartphone IPSS Q5	1581	2.30 (1.67)	0-5
Smartphone IPSS Q6	1580	1.27 (1.46)	0-5
Smartphone IPSS Q7	1581	1.28 (1.16)	0-7
Smartphone Total IPSS	1581	11.03 (7.77)	0-35
Smartphone QOL	1546	2.77 (1.37)	0-6
Mild LUTS^c^	668 (42.25%)	--	--
Moderate LUTS	643 (40.67%)	--	--
Severe LUTS	270 (17.08%)	--	--

^a^IPSS: International Prostate Symptom Score

^b^QOL: quality of life

^c^LUTS: lower urinary tract symptoms

### Validation With Paired *t* Test

There were no differences in the overall total scores of the IPSS (*P*=.289) and each item of the IPSS (*P* values =.157-1.000) between the paper version of the IPSS and smartphone version of the IPSS (Table 2). In the mild LUTS group, the total score of the IPSS (*P*=.752) and each item of the IPSS (*P* values =.157-1.000) showed no differences between the paper version and the smartphone version of the IPSS (Table 3). In the moderate LUTS group, the total score of the IPSS (*P*=.432) and each item of the IPSS (*P* values =.103-1.000) showed no differences between the paper version and the smartphone version of the IPSS (Table 4). In the severe LUTS group, the total score of the IPSS (*P*=.083) and each item of the IPSS (*P* values =.158-1.000) showed no differences between the paper version and the smartphone version of the IPSS (Table 5).

**Table 2:** Paired *t* test between paper and smartphone IPSS^a^.

	Paper, mean (SD)	Smartphone, mean (SD)	*P* value
IPSS Q1	1.69 (1.61)	1.68 (1.61)	.157
IPSS Q2	1.57 (1.42)	1.57 (1.42)	.166
IPSS Q3	1.63 (1.54)	1.63 (1.54)	.435
IPSS Q4	1.32 (1.42)	1.32 (1.42)	.578
IPSS Q5	2.30 (1.66)	2.30 (1.66)	.223
IPSS Q6	1.27 (1.46)	1.27 (1.46)	1.000
IPSS Q7	1.28 (1.15)	1.28 (1.16)	.317
Total IPSS	11.04 (7.77)	11.03 (7.77)	.289
IPSS QOL^b^	2.77 (1.38)	2.77 (1.37)	.180

^a^IPSS: International Prostate Symptom Score

^b^QOL: quality of life

**Table 3:** Paired *t* test between paper and smartphone IPSS^a^ in mild LUTS^b^.

	Paper, mean (SD)	Smartphone, mean (SD)	*P* value
IPSS Q1	0.53 (0.71)	0.53 (0.71)	1.000
IPSS Q2	0.60 (0.71)	0.60 (0.71)	.706
IPSS Q3	0.53 (0.67)	0.53 (0.66)	.157
IPSS Q4	0.39 (0.62)	0.40 (0.62)	.257
IPSS Q5	0.99 (0.92)	0.99 (0.93)	1.000
IPSS Q6	0.37 (0.62)	0.38 (0.620	.706
IPSS Q7	0.73 (0.77)	0.72 (0.77)	.180
Total IPSS	4.13 (2.09)	4.13 (2.10)	.752
IPSS QOL^c^	1.86 (1.10)	1.86 (1.10)	1.000

^a^IPSS: International Prostate Symptom Score

^b^LUTS: lower urinary tract symptoms

^c^QOL: quality of life

**Table 4:** Paired *t* test between paper and smartphone IPSS^a^ in moderate LUTS^b^.

	Paper, mean (SD)	Smartphone, mean (SD)	*P* value
IPSS Q1	1.93 (1.30)	1.93 (1.30)	.318
IPSS Q2	1.82 (1.14)	1.81 (1.14)	.103
IPSS Q3	1.87 (1.19)	1.87 (1.20)	.631
IPSS Q4	1.53 (1.19)	1.53 (1.19)	.655
IPSS Q5	2.75 (1.31)	2.74 (1.30)	.127
IPSS Q6	1.36 (1.15)	1.35 (1.15)	.819
IPSS Q7	1.34 (1.01)	1.34 (1.01)	1.000
Total IPSS	12.59 (3.30)	12.58 (3.32)	.432
IPSS QOL^c^	2.99 (1.02)	2.99 (1.01)	.318

^a^IPSS: International Prostate Symptom Score

^b^LUTS: lower urinary tract symptoms

^c^QOL: quality of life

**Table 5:** Paired *t* test between paper and smartphone IPSS^a^ in severe LUTS^b^.

	Paper, mean (SD)	Smartphone, mean (SD)	*P* value
IPSS Q1	3.96 (1.12)	3.95 (1.15)	.318
IPSS Q2	3.38 (1.33)	3.38 (1.33)	1.000
IPSS Q3	3.81 (1.26)	3.81 (1.26)	1.000
IPSS Q4	3.09 (1.49)	3.09 (1.49)	1.000
IPSS Q5	4.46 (0.79)	4.46 (0.79)	.158
IPSS Q6	3.29 (1.51)	3.29 (1.52)	1.000
IPSS Q7	2.51 (1.28)	2.51 (1.28)	1.000
Total IPSS	24.45 (3.58)	24.44 (3.58)	.083
IPSS QOL^c^	4.45 (0.81)	4.43 (0.82)	.318

^a^IPSS: International Prostate Symptom Score

^b^LUTS: lower urinary tract symptoms

^c^QOL: quality of life

### Reliability Test With Interclass Correlation Coefficient (ICC) Test

Reliability was assessed using the ICC and a two-way random effect model, assuming a single measurement and absolute agreement. A significant correlation was noted in the total group (ICC=.935, *P*<.001). The mild, moderate, and severe LUTS groups also showed significant correlations (ICCs =.616, .549, and .548 respectively, all *P* values <.001) (Table 6).

**Table 6:** Reliability test of paper and smartphone versions of the IPSS^a^.

	Interclass correlation coefficient	95% CI		*P* value
		Lower	Upper	
Total	.935	0.927	0.941	<.001
Mild LUTS^b^	.616	0.571	0.659	<.001
Moderate LUTS	.549	0.492	0.602	<.001
Severe LUTS	.548	0.462	0.625	<.001

^a^IPSS: International Prostate Symptom Score

^b^LUTS: lower urinary tract symptoms

### Compliance

For compliance, we created two questions that asked, “Which method would you be willing to use to complete the assessment more easily?” and “Which method would you prefer?” In the examination of feasibility, 760 (48.07%, 760/1581) participants replied that the smartphone version was more feasible, 420 (26.56%, 420/1581) participants replied that the paper version was more feasible, and 301 (19.03%, 301/1581) participants replied that both of the versions were feasible. With regard to preference, 820 (51.86%, 820/1581) participants preferred the smartphone version, 320 participants (20.24%, 320/1581) preferred the paper version, and 356 (22.51%, 356/1581) participants showed no preference. For the two questionnaires, the results showed significant differences according to age (*P*<.001).

## Discussion

### Principal Findings

**Figure 2:**
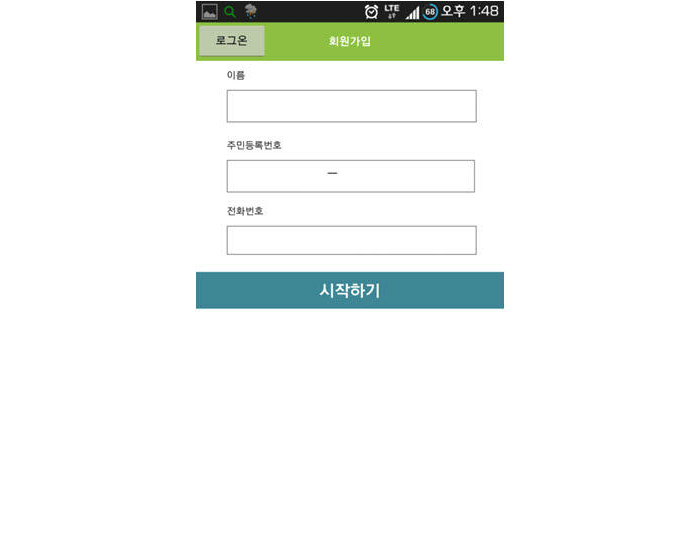
Screenshot of the IPSS Android smartphone application. IPSS: International Prostate Symptom Score.

**Figure 3:**
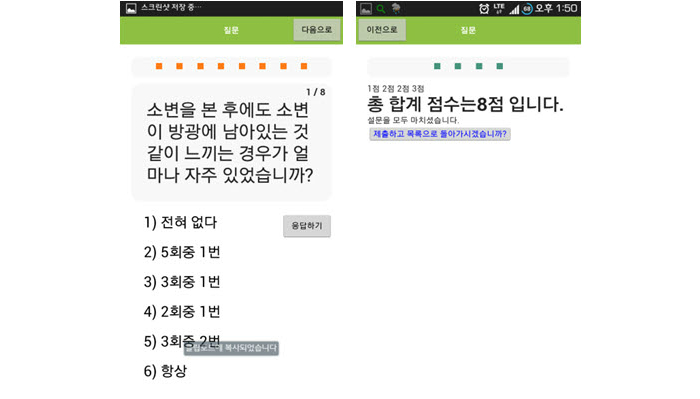
IPSS question 1 and ending screen in the smartphone application. IPSS: International Prostate Symptom Score.

**Figure 4:**
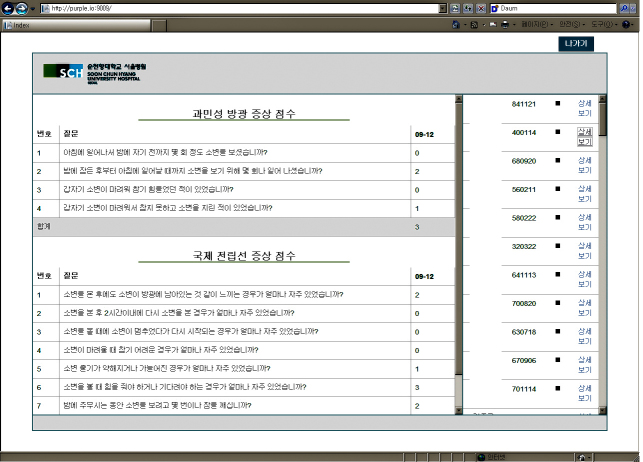
Registered IPSS scores are automatically transferred to main server. IPSS: International Prostate Symptom Score.

The objective of this study was to compare two different methods for diagnostic assessment via screening questionnaires. The overall hypothesis of this study was that a smartphone application of IPSS would show the same efficacy in real clinical use as a diagnostic questionnaire. This study was the first to show the clinical use of a smartphone questionnaire application in the field of urology (Figures 2-4).

The recent remarkable increase in the adoption of smartphones enables not only easy communication, but also the possibility of utilizing devices in diverse settings, including health care. There have been several studies using smartphone applications of sleep questionnaires, including the Epworth Sleepiness Scale, the Berlin questionnaire, and the STOP BANG questionnaire [10]. In psychiatric disease and serious mental illness, smartphone-based questionnaires for monitoring have shown better compliance than other types of questionnaires. Ambulatory monitoring of symptoms using smartphone applications represents a feasible and valid way of assessing psychotic status for research and clinical management [11,16]. Smartphone application questionnaires have also been used in a cardiac rehabilitation population for measuring physical activity with demonstrated validity and reliability [12].For clinical application as a medical device, a smartphone-based application has been used for actigraphy recording and audio recording in sleep disorders [10]. However, none were statistically validated by a comparison with the paper version of the questionnaire by a non-inferior trial with the hypothesis that the smartphone version of the questionnaire could be as effective as the paper version of the questionnaire. Little is known about the strengths and limitations of smartphone application questionnaires in the screening or monitoring of diseases, especially in the field of urology. Only one study has shown the validity and reliability of a smartphone application for the assessment of penile deformity in Peyronie’s disease [17]. There are several urologic applications such as “Bladder Pal”, “Prostate Pal”, “Get Bladder Fit”, and “UroApp”. Prostate Pal provides an easily accessible tool for recording intake and output, AUA score, and PSA level, but this application showed no validity.

The main issue in mobile research is ensuring the security of patient data. It is an important problem and it has been argued that it is not safe for health care clinicians to have access to patient information from a handheld device [18]. This is the main reason that we designed this Web-based application to ensure that patient information and clinical data were not saved on patients’ own smartphones, but transferred directly to the main server. Administration of the server was only performed by clinicians and, after data were transferred to the hospital server, the records were removed.

The merits of the clinical use of a smartphone application are the possibility of yielding a great number of data points, requiring less time, and more positive compliance by participants than conventional methods, including paper, telephone, and email methods. From a technical view, a smartphone application has computing power, a touch screen, third-party application development and distribution, and high-speed data transfer.

Smartphones can effectively use real-time upload and backup, which can prevent data loss [19,20]. With regard to feasibility, users believed smartphones were easier to use than a conventional paper system in a study of an Android-based application for men’s health [19,21]. In terms of time consumption, a smartphone application has merits over paper questionnaires and Web-based research [12,22,23]. Moreover, increased access and availability to smartphone communication increases the potential for large scale surveys in population-based studies.

We developed this application for the Android operating system. The two main discriminatory factors for determining the operating system were the widespread popularity and homogeneity of the smartphone hardware. We adopted the Android operating system because the number of its users is rapidly growing. With regard to homogenous hardware safety, the iPhone may be a better option.

There are regulatory barriers to the clinical application of smartphone-based medical devices, but smartphone-based questionnaires such as our application are considered Class I devices by the FDA [24,25]. Class I devices represent general devices that are not designed for use in supporting or sustaining life nor are of considerable importance in preventing impairment to human life and have the least demanding restrictions of the three FDA device classes. In recent guidelines, the FDA classified Medical Device Data System (MDDS) software as Class I, because it transfers, stores, converts, or displays medical device data without providing analysis, alarms, or active patient monitoring. This latest set of MDDS guidelines came after the European Commission decision that most applications would be classified under Class I. Our application is a simple substitute for a paper-based questionnaire and does not involve analysis of data, alarms, or active patient monitoring.

### Limitations

This study has several limitations. First, there could be selection bias in this study. Some patients reported a mild negative reaction to the questionnaire method. It is possible that smartphone assessment is less suitable in certain subgroups of patients. Some groups, including older-aged people, may use this mobile technology less than others and find it to be a barrier. However, it is estimated that 80-90% of the population will have a smartphone within 10 years [26]. Considering the rapid increase in the use of smartphones, older-aged people will increasingly come to be familiar with smartphones.

There is also skepticism over the possible differences between self-reported measures and clinician-based ratings. The IPSS was originally developed as a self-reported questionnaire and self-report measures may be time and cost-saving methods.

Although we demonstrated the validity of a smartphone application of the IPSS by quantitative analysis, concerns still remain regarding the quality of the analysis, including data recording, data entry, reliability, time consumption, and costs.

Our application does not contain open-ended questions, and therefore we did not examine issues of quality, except for compliance and satisfaction rates. A more streamlined graphical and colorful user interface may lead to better compliance for participants for both screening and monitoring.

We divided patients into three groups based on the severity of their symptoms as ascertained by their QOL scores: mild (0-2), moderate (3-4), and severe (5-6). This classification was arbitrary. We did not consider sociodemographic factors of patients that could impact the development of LUTS. Nevertheless, to our knowledge, this is the first study to demonstrate the validity of a smartphone version of the IPSS by scientific methods.

### Conclusions

In summary, a smartphone application of the IPSS has validity and reliability, which means that it is not inferior to the paper version of the IPSS. Further research is needed to test its efficacy in the monitoring of LUTS. Future studies are needed to demonstrate its role in consecutive monitoring and also its usefulness in cost savings and data collection.

## Abbreviations

BPH: benign prostatic hyperplasia
ICC: intraclass correlation coefficient
IPSS: International Prostate Symptom Score
LUTS: lower urinary tract symptoms
MDDS: Medical Device Data System

## References

[1] Quek KF, Loh CS, Low WY, Razack AH (2001). Quality of life assessment before and after transurethral resection of the prostate in patients with lower urinary tract symptoms. World J Urol.

[2] Tsang KK, Garraway WM (1993). Impact of benign prostatic hyperplasia on general well-being of men. Prostate.

[3] Barry MJ (2001). Evaluation of symptoms and quality of life in men with benign prostatic hyperplasia. Urology.

[4] Roehrborn CG (2011). Male lower urinary tract symptoms (LUTS) and benign prostatic hyperplasia (BPH). Med Clin North Am.

[5] Barry MJ, Cockett AT, Holtgrewe HL, McConnell JD, Sihelnik SA, Winfield HN (1993). Relationship of symptoms of prostatism to commonly used physiological and anatomical measures of the severity of benign prostatic hyperplasia. J Urol.

[6] Barry MJ, Fowler FJJr, O'Leary MP, Bruskewitz RC, Holtgrewe HL, Mebust WK (1995). Measuring disease-specific health status in men with benign prostatic hyperplasia. Measurement Committee of The American Urological Association. Med Care.

[7] Tomlinson M, Solomon W, Singh Y, Doherty T, Chopra M, Ijumba P, Tsai AC, Jackson D (2009). The use of mobile phones as a data collection tool: a report from a household survey in South Africa. BMC Med Inform Decis Mak.

[8] Nielsen (2010). Smartphones to overtake feature phones in US by 2011.

[9] Ozdalga E, Ozdalga A, Ahuja N (2012). The smartphone in medicine: a review of current and potential use among physicians and students. J Med Internet Res.

[10] Behar J, Roebuck A, Domingos JS, Gederi E, Clifford GD (2013). A review of current sleep screening applications for smartphones. Physiol Meas.

[11] Palmier-Claus JE, Ainsworth J, Machin M, Barrowclough C, Dunn G, Barkus E, Rogers A, Wykes T, Kapur S, Buchan I, Salter E, Lewis SW (2012). The feasibility and validity of ambulatory self-report of psychotic symptoms using a smartphone software application. BMC Psychiatry.

[12] Pfaeffli L, Maddison R, Jiang Y, Dalleck L, Löf M (2013). Measuring physical activity in a cardiac rehabilitation population using a smartphone-based questionnaire. J Med Internet Res.

[13] Bowling A (2005). Mode of questionnaire administration can have serious effects on data quality. J Public Health (Oxf).

[14] Choi HR, Chung WS, Shim BS (1996). Translation validity and reliability of I-PSS Korean version. Korean J Urol.

[15] Cam K, Akman Y, Cicekci B, Senel F, Erol A (2004). Mode of administration of international prostate symptom score in patients with lower urinary tract symptoms: physician vs self. Prostate Cancer Prostatic Dis.

[16] Ainsworth J, Palmier-Claus JE, Machin M, Barrowclough C, Dunn G, Rogers A, Buchan I, Barkus E, Kapur S, Wykes T, Hopkins RS, Lewis S (2013). A comparison of two delivery modalities of a mobile phone-based assessment for serious mental illness: native smartphone application vs text-messaging only implementations. J Med Internet Res.

[17] Hsi RS, Hotaling JM, Hartzler AL, Holt SK, Walsh TJ (2013). Validity and reliability of a smartphone application for the assessment of penile deformity in Peyronie's disease. J Sex Med.

[18] Haynes CL, Cook GA, Jones MA (2007). Legal and ethical considerations in processing patient-identifiable data without patient consent: lessons learnt from developing a disease register. J Med Ethics.

[19] Zhang S, Wu Q, van Velthoven MH, Chen L, Car J, Rudan I, Zhang Y, Li Y, Scherpbier RW (2012). Smartphone versus pen-and-paper data collection of infant feeding practices in rural China. J Med Internet Res.

[20] Yu P, de Courten M, Pan E, Galea G, Pryor J (2009). The development and evaluation of a PDA-based method for public health surveillance data collection in developing countries. Int J Med Inform.

[21] Rajput ZA, Mbugua S, Amadi D, Chepngeno V, Saleem JJ, Anokwa Y, Hartung C, Borriello G, Mamlin BW, Ndege SK, Were MC (2012). Evaluation of an Android-based mHealth system for population surveillance in developing countries. J Am Med Inform Assoc.

[22] Rabin C, Bock B (2011). Desired features of smartphone applications promoting physical activity. Telemed J E Health.

[23] Granholm E, Ben-Zeev D, Link PC, Bradshaw KR, Holden JL (2012). Mobile Assessment and Treatment for Schizophrenia (MATS): a pilot trial of an interactive text-messaging intervention for medication adherence, socialization, and auditory hallucinations. Schizophr Bull.

[24] (2011). US Food and Drug Administration.

[25] (2013). US Food and Drug Administration.

[26] Boulos MN, Wheeler S, Tavares C, Jones R (2011). How smartphones are changing the face of mobile and participatory healthcare: an overview, with example from eCAALYX. Biomed Eng Online.

